# Effects of exogenous glucagon-like peptide-2 and distal bowel resection on intestinal and systemic adaptive responses in rats

**DOI:** 10.1371/journal.pone.0181453

**Published:** 2017-07-24

**Authors:** Sarah W. Lai, Elaine de Heuvel, Laurie E. Wallace, Bolette Hartmann, Jens J. Holst, Mary E. Brindle, Prasanth K. Chelikani, David L. Sigalet

**Affiliations:** 1 Department of Surgery, Faculty of Medicine, University of Calgary, Calgary, Alberta, Canada; 2 Department of Biomedical Sciences, Novo Nordisk Foundation Center for Basic Metabolic Research, University of Copenhagen, The Panum Institute, Copenhagen, Denmark; 3 Department of Production Animal Health, Faculty of Veterinary Medicine, University of Calgary, Calgary, Alberta, Canada; 4 Department of Surgery, Sidra Medical and Research Center and Weil Cornell Medical College, Doha, Qatar; University of Texas Medical Branch, UNITED STATES

## Abstract

**Objective:**

To determine the effects of exogenous glucagon-like peptide-2 (GLP-2), with or without massive distal bowel resection, on adaptation of jejunal mucosa, enteric neurons, gut hormones and tissue reserves in rats.

**Background:**

GLP-2 is a gut hormone known to be trophic for small bowel mucosa, and to mimic intestinal adaptation in short bowel syndrome (SBS). However, the effects of exogenous GLP-2 and SBS on enteric neurons are unclear.

**Methods:**

Sprague Dawley rats were randomized to four treatments: Transected Bowel (TB) (n = 8), TB + GLP-2 (2.5 nmol/kg/h, n = 8), SBS (n = 5), or SBS + GLP-2 (2.5 nmol/kg/h, n = 9). SBS groups underwent a 60% jejunoileal resection with cecectomy and jejunocolic anastomosis. All rats were maintained on parenteral nutrition for 7 d. Parameters measured included gut morphometry, qPCR for hexose transporter (SGLT-1, GLUT-2, GLUT-5) and GLP-2 receptor mRNA, whole mount immunohistochemistry for neurons (HuC/D, VIP, nNOS), plasma glucose, gut hormones, and body composition.

**Results:**

Resection increased the proportion of nNOS immunopositive myenteric neurons, intestinal muscularis propria thickness and crypt cell proliferation, which were not recapitulated by GLP-2 therapy. Exogenous GLP-2 increased jejunal mucosal surface area without affecting enteric VIP or nNOS neuronal immunopositivity, attenuated resection-induced reductions in jejunal hexose transporter abundance (SGLT-1, GLUT-2), increased plasma amylin and decreased peptide YY concentrations. Exogenous GLP-2 attenuated resection-induced increases in blood glucose and body fat loss.

**Conclusions:**

Exogenous GLP-2 stimulates jejunal adaptation independent of enteric neuronal VIP or nNOS changes, and has divergent effects on plasma amylin and peptide YY concentrations. The novel ability of exogenous GLP-2 to modulate resection-induced changes in peripheral glucose and lipid reserves may be important in understanding the whole-body response following intestinal resection, and is worthy of further study.

## Introduction

Neonatal short bowel syndrome (**SBS**) occurs after massive resection of the small bowel for various pathologies, including necrotizing enterocolitis, atresias and midgut volvulus. SBS may lead to intestinal failure when there is inadequate bowel length necessary for survival, resulting in severe diarrhea, electrolyte abnormalities and failure to thrive. Initial management involves correction of electrolyte, acid base and fluid imbalances, along with nutritional support. Total parenteral nutrition (**TPN**) is used to provide calories until intestinal adaptation occurs. Intestinal adaptation is defined by increases in mucosal surface area for digestion and absorption, as well as functional modifications to bowel motility, digestive enzyme activity and nutrient transporter expression [1, 2]. Intraluminal nutrients are potent stimuli for adaptation, triggering the release of enteric hormones such as glucagon-like peptide-2 (**GLP-2**) [3, 4].

Glucagon-like peptide-2 is a 33 amino acid proglucagon-derived gut hormone that is synthesized and secreted from enteroendocrine L-cells of the terminal ileum and colon [3, 5]. GLP-2 has been implicated as a key player in the adaptive process and a potential therapy for SBS [6]. Treatment with exogenous GLP-2 has been shown to be intestinotrophic with effects on mucosal surface area, proliferation, apoptosis, permeability, motility and blood flow [7, 8]. GLP-2 acts through a GLP-2 receptor (**GLP-2R**) that is found primarily in the gut, but also in the brain. In the gastrointestinal tract, GLP-2R has been reported to be localized to subepithelial myofibroblasts and enteric neurons, but not intestinal epithelial cells [9–14].

There is substantial evidence suggesting that the actions of GLP-2 on mucosal adaptation are signalled, in part, by the enteric nervous system (**ENS**). GLP-2R have been colocalized to ENS neurons expressing choline-acetyltransferase, vasoactive intestinal peptide (**VIP**) and nitric oxide synthase (**NOS**) [15, 16]. It has been suggested that GLP-2 promotes intestinal adaptation by inhibiting excitatory neurons and stimulating inhibitory neurons, resulting in increased mesenteric vascular flow and reduced intestinal motility [15–18]. We have previously shown that GLP-2 treatment alters submucosal neuronal populations *in vitro* by increasing the proportion of VIP and neuronal NOS (**nNOS**) expression, and stimulates VIP immunopositivity *in vivo* in colitis models [19, 20].

In addition to well-described intestinotrophic effects, murine studies indicate that central GLP-2 action through pro-opiomelanocortin neurons decreases hepatic glucose production and increases glucose clearance by peripheral tissues [14]. Infusion of GLP-2 in humans with SBS has been shown to have no effect on fasting or post-prandial blood glucose levels; thus, the role of GLP-2 in glycemic control remains unclear [21]. Importantly, the effects of exogenous GLP-2 and bowel resection, alone and in combination, on morphology of the submucosal and myenteric neuronal plexuses of the gut, as well as glucose homeostasis, have yet to be determined.

In this study, using a model of fasting rats maintained solely on TPN, we determined whether bowel resection and GLP-2 treatment induce adaptive changes in the intestinal mucosa and ENS, and assessed changes in circulating glucose, gut hormones and body composition. We hypothesized that exogenous GLP-2 recapitulates the native adaptive response after massive distal resection via an increase in the proportion of VIP and nNOS expressing neurons. We also hypothesized that both exogenous GLP-2 and resection would modulate the circulating concentrations of other gut peptides and glucose, as well as alter body composition.

## Materials and methods

### Animals

Animal studies were conducted with the approval of the Animal Care Committee at the University of Calgary (Study #AC12-0103) in strict accordance with the Canadian Council for Animal Care. Male Sprague Dawley rats (Charles River, Trois-Rivières, QC) weighing 225 to 275 g were housed in cages with free access to chow and water. Animals were acclimatized to their environment for 5 d prior to experimentation under standardized temperature, humidity and 12 h light-dark cycles.

### Surgical procedures

**Fig 1:**
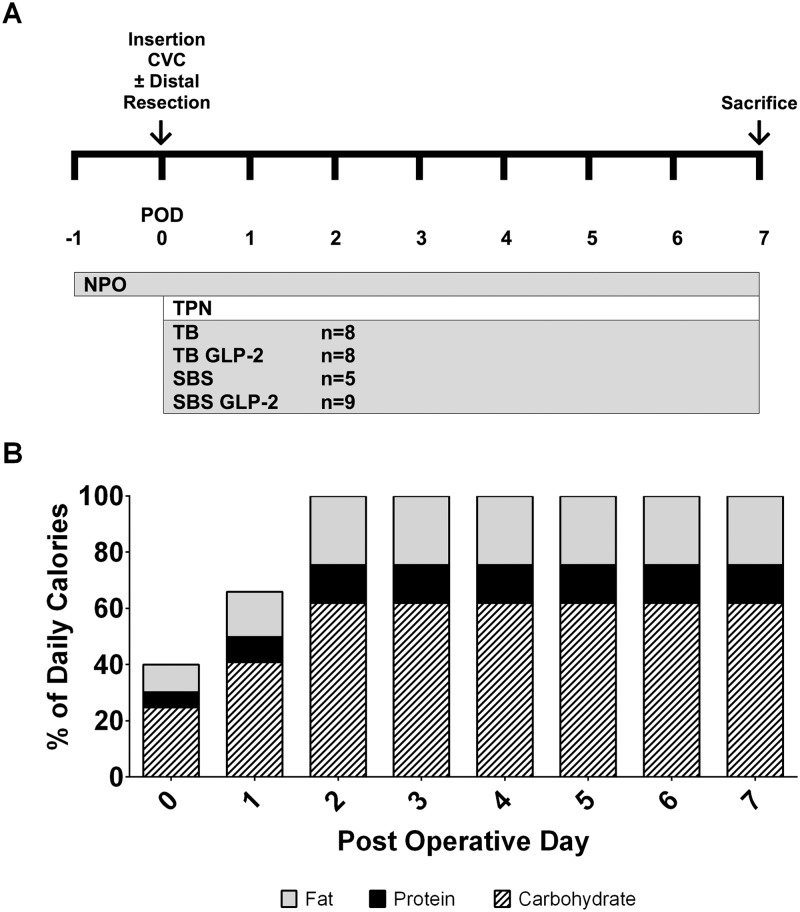
Timeline for experimental procedures. Rats were kept NPO from one day before surgery until the end of experimentation, and maintained on TPN for 7 d after surgery for bowel transection or resection (A). GLP-2 rats were given recombinant human GLP-2 (1–33) mixed into the daily volume of TPN as a continuous infusion (2.5 nmol/kg/h). Fat, protein and carbohydrate content in TPN were advanced step-wise to reach 100% of daily calories (250 kcal/kg/d) by POD 2 (B). Abbreviations: *nil per os* (**NPO**), post-operative day (**POD**).

The timeline of experimental procedures is shown in Fig 1. Thirty rats were randomized to four treatment groups: (1) transected bowel (**TB**, n = 8), (2) TB GLP-2 (n = 8), (3) short bowel syndrome (SBS, n = 5), and (4) SBS GLP-2 (n = 9). Animals received either a sham laparotomy with bowel transection (TB) or a 60% jejunoileal resection with cecectomy (SBS), and were subsequently treated with or without GLP-2 (human recombinant GLP-2 (1–33); NPS Pharmaceuticals, Mississauga, ON). Surgical procedures were adapted from previously published techniques [22, 23]. Animals were fasted for 24 h prior to surgery until sacrifice, with free access to water. All procedures were performed using aseptic technique with the aid of surgical telescopes (Designs for Vision, Ronkonkoma, NY) under isoflurane anesthesia (1.5%; Pharmaceutical Partners of Canada, Richmond Hill, ON) and oxygen (0.6 L/min). Subcutaneous cefazolin (50 mg/kg; Pharmaceutical Partners of Canada, Richmond Hill, ON) was administered for surgical prophylaxis prior to skin incision. A silastic central venous catheter (**CVC**, 1.65 mm outer diameter, 0.76 mm inner diameter; Dow Corning, Midland, MI) was tunnelled from the back, anchored using a tethered metal sheath (Harvard Apparatus Canada, Saint-Laurent, QC) and 4–0 silk (Ethicon, Somerville, NJ), then inserted into the right jugular vein. The CVC was attached to a standard free swivel device (Harvard Apparatus Canada, Saint-Laurent, QC) and infused with heparinized saline (1.5 U heparin/mL normal saline, 1 mL/h; LEO Pharma Inc, Thornhill, ON). After laparotomy, the jejunum was transected 40 cm from the ligament of Treitz. For TB groups, the jejunum was reanastomosed immediately. For SBS animals, an approximate 60% jejunoileal resection with cecectomy was performed from the jejunal transection to 1 cm distal to the cecum, followed by a jejunocolic anastomosis. The length of resected bowel was not measured, as the length of remaining jejunum was kept constant. An interrupted Gambee appositional technique with 7–0 polypropylene (Ethicon, San Lorenzo, Puerto Rico) was used for all anastomoses [24]. Mesenteric vessels were controlled using 4–0 silk ties, and the mesenteric defect was closed using 7–0 polypropylene. Hemostasis was ensured and the animal was resuscitated with 5 mL of warm intraperitoneal saline prior to a layered abdominal closure using 4–0 polyglactin (Ethicon, Somerville, NJ). Animals were placed in individual cages and the CVC was reconnected for continuous infusion of TPN.

### Total parenteral nutrition

Rats received nutritional support consisting of 14% protein, 62% carbohydrate and 24% fat (250 kcal/kg/d) as a continuous infusion of TPN. TPN solutions were adapted from previously published protocols and prepared aseptically in advance, using 8.5% Travasol, 70% dextrose, 20% Intralipid, commercial multivitamins and trace elements (Baxter Corporation, Toronto, ON) [6, 25]. Heparin (1.5 U/mL) and cefazolin (50 mg/kg/d) were added to the TPN for prophylaxis until sacrifice. GLP-2 treated animals were given recombinant human GLP-2 (1–33) as a continuous infusion (2.5 nmol/kg/h) mixed into the daily volume of TPN using a PHD Ultra Syringe Pump (Harvard Apparatus Canada, Saint-Laurent, QC) to mimic GLP-2 levels attained from resected animals in previous studies [6]. Animals were maintained with 40% of their daily caloric requirements via TPN for the first 12 h, then advanced step-wise to 66% on POD 1, and 100% on POD 2 until sacrifice on POD 7 to mitigate risks of hyperglycemia and hypertriglyceridemia (Fig 1B).

### Tissue sampling

On POD 7, rats received intravenous injection of 5-bromo-2’-deoxyuridine (**BrdU**, 100 mg/kg; Sigma-Aldrich, St. Louis, MO) 1 h prior to sacrifice for further immunohistochemical analysis. Animals were anesthetised with inhaled isoflurane (1.5%), weighed and sacrificed by exsanguination in the morning on POD 7. Prior to death, blood was collected by direct cardiac puncture and glucose concentration was immediately determined using a hand-held Accu-Chek Glucose Meter (Roche Diagnostics, Laval, QC). The remaining sample was immediately aliquoted into tubes containing diprotin-A (0.1 mM; Sigma-Aldrich Inc, St. Louis, MO) and aprotinin (500 KU/mL blood; Bayer Inc, Toronto, ON) for plasma GLP-2 quantification, and tubes containing a cocktail of dipeptidyl peptidase-IV inhibitor (10 μL/mL blood; Millipore Corporation, Billerica, CA) and protease inhibitors (10 μL/mL blood; Sigma, St. Louis, MO) for other hormone assays. Samples were centrifuged at 2500 g for 10 min at 4°C and the plasma stored at -80°C until analysis. Plasma GLP-2 concentrations were measured using a GLP-2 (1–33) specific radioimmunoassay with intra-assay coefficient of variation of 5%, as previously described [26]. Plasma concentrations of active GLP-1, insulin, total peptide YY (**PYY**) and amylin were measured using a customized Milliplex rat gut hormone panel on a Luminex Bio-Plex 200 platform with intra-assay coefficient of variation 3.8% to 10.6% (Millipore, Luminex Co, Austin, TX).

Gross morphometry was quantified by removing remaining bowel from the ligament of Treitz to the rectum, excluding the mesentery. The remnant jejunum and colon, devoid of enteric contents, were weighed and midpoint circumference measured. Representative segments of the jejunum (proximal 10 cm distal to ligament of Treitz) and colon (proximal 10 cm after discarding 0.5 cm distal to anastomosis) were fixed in 10% formalin (EMD Chemicals Inc, Gibbstown, NJ) for histology, placed into RNALater buffer (Ambion Inc, Austin TX) and stored at -80°C for semi-quantitative polymerase chain reaction (**qPCR**), or fixed in Zamboni’s solution (Newcomer Supply, Middleton, WI) overnight at 4°C for whole mount immunohistochemistry.

### Body composition

After tissue collection, carcasses were placed individually into plastic bags, sealed and frozen at -20°C for quantitative magnetic resonance measurements of fluid, fat and lean body mass. Specimens were thawed to room temperature and scanned over 2 min for body composition using the Minispec LF110 Body Composition Analyzer (Bruker Ltd, Milton, ON) following previously published procedures [27]. Given the short duration of treatment intervention, the changes in individual body compartments were expressed as a percentage of the total.

### Histology and immunohistochemistry

Paraffin sections (6 μm) of jejunum and colon were stained with hematoxylin and eosin. Measurements of villus height, villus width, crypt width and muscularis propria thickness were recorded from 10 representative, well-oriented villus/crypt units, and jejunal mucosal-to-serosal amplification ratios and mucosal surface areas calculated as previously described [28]. Crypt cell proliferation was quantified using BrdU as a marker of active cell division, adapted from previous protocols [29]. Deparaffinised sections were incubated in citric acid (0.01 M, pH 6.0; Fisher Scientific, Fairlawn, NJ) for 90 s, 1% TritonX100 (Fisher Scientific, Orange County, NY) in phosphate buffered saline (Sigma-Aldrich Inc, St. Louis, MO) for 10 min at room temperature, treated with serial concentrations of HCl (1 N for 10 min on ice, 2 N for 10 min at room temperature, 2 N for 20 min at 37°C; VWR International, West Chester, PA), then borate buffer (0.1 M, pH 8.5; Fisher Scientific, Fairlawn, NJ) for 12 min at room temperature. Samples were incubated in 10% normal horse serum (Sigma-Aldrich, St. Louis, MO) for 5 min, then with anti-BrdU-biotin (#ab2284, 1:250; Abcam, Toronto, ON) overnight, followed by 3% hydrogen peroxide (Sigma-Aldrich, St. Louis, MO) for 20 min, detected with ABC-kit (Vector Laboratories, Burlingame, CA) for 30 min, and finally counterstained with hematoxylin for nuclei; all steps at room temperature. Ten representative, well-oriented crypts were inspected for BrdU-stained nuclei and total nuclei to determine the crypt cell proliferation index (CCPI) by an observer blinded to the treatment groups. Measurements and images were taken using an Axiovert S100TV microscope (Carl Zeiss, Thornwood, NY) and DFC490 digital imaging system (Leica Microsystems, Vertrieb, Germany).

### Semi-quantitative polymerase chain reaction (qPCR)

qPCR was performed for sodium-glucose linked transporter-1 (**SGLT-1**), glucose transporter-2 and 5 (**GLUT-2**, **GLUT-5**), and GLP-2R in the jejunum, using 18S ribosomal RNA as the endogenous control, following our published procedures [30]. Total mRNA was isolated from each bowel segment using QIAzol Lysis Reagent and miRNeasy Mini Kit (Qiagen Inc, Toronto, ON) according to the manufacturer’s instructions, and quantified using a nanoVette DU 730 spectrophotometer (Beckman Coulter Inc, Indianapolis, IN). Samples were diluted to a uniform concentration of 250 ng/μL with RNAse free water (Qiagen Inc, Toronto, ON), treated with DNAse (Invitrogen, Burlington, ON) and ethylenediaminetetraacetic acid (Invitrogen, Burlington, ON), then reverse transcribed using SuperScript II (Invitrogen, Burlington, ON) in a Mastercycler Pro Ep Realplex Thermocycler (Eppendorf, Mississauga, ON) as previously described [30]. qPCR was performed using Power SYBR Green Master Mix (Applied Biosystems Inc, Burlington, ON) in a Mastercycler Ep Gradient Thermocycler Detection System (Eppendorf, Mississauga, ON) using specific primers for SGLT-1, GLUT-2, GLUT-5, GLP-2R and 18S after optimization for amplification efficiency (90% to 110%). Primer sets were synthesized (University Core DNA Services, Calgary, AB) based on sequences listed in Table 1. Samples were run in duplicate under the following conditions: initial denaturation (50°C for 2 min, 95°C for 10 min), 40 cycles of amplification (denaturation at 95°C for 15 s, primer-specific annealing temperature between 55°C to 65°C for 1 min), and final melting (95°C for 15 s, 60°C for 15 s, ramping for 20 min up to 95°C for 15 s). Target gene abundance was calculated as a relative fold-change based on the ΔΔCt method [31].

**Table 1:** Target and endogenous control gene primer sequences and annealing temperatures for qPCR.

Primer	Sequence	Annealing Temperature (°C)	Species	Accession #
SGLT-1	(F) CCAAGCCCATCCCAGACGTACACC	55	Rat	NM_013033.2
	(R) CTTCCTTAGTCATCTTCGGTCCTT			
GLUT-2	(F) TTTGCAGTAGGCGGAATGG	60	Rat	NM_012879.2
	(R) GCCAACATGGCTTTGATCCTT			
GLUT-5	(F) TGCAGAGCAACGATGGAGAAA	59	Rat	NM_031741.1
	(R) ACAGCAGCGTCAGGGTGAAG			
GLP-2R	(F) ACCTTGCAGCTGATGTACAC	65	Rat	NM_021848.1
	(R) CAGCCAGAACTTTCAGGATG			
18S	(F) ACGGACCAGAGCGAAAGCAT	60	Rat	M11188.1
	(R) TGTCAATCCTGTCCGTGTCC			

Abbreviations: forward (**F**), reverse (**R**).

### Neuronal whole mount immunohistochemistry

After overnight incubation in Zamboni’s fixative at 4°C, tissues were dissected to isolate the submucosal and myenteric plexuses separately, and costained with HuC/D-VIP or HuC/D-nNOS as adapted from previous methods [19]. Whole mounts were incubated in monoclonal anti-HuC/D (1:100, #A-21271; Molecular Probes, Eugene, OR) and polyclonal anti-VIP (1:400, #B34-1; Cedarlane, Burlington, ON) or polyclonal anti-nNOS (1:800, #B220-1; Cedarlane, Burlington, ON) overnight at 4°C, detected with polyclonal anti-mouse 488 (1:100, #715-545-151; Jackson ImmunoResearch, West Grove, PA) and polyclonal anti-rabbit Cy3 (1:1000, #111-165-144; Jackson ImmunoResearch, West Grove, PA) for 2 h at room temperature, and mounted onto slides with FluorSave (Calbiochem, EMD Chemicals Inc, Gibbstown, NJ). Five random high powered fields (**HPF**) from each specimen were assessed for ENS morphology. The number of ganglia per HPF and the total number of HuC/D staining neurons per ganglia were quantified. The proportion of neurons that costained for VIP or nNOS was deduced by dividing the number of VIP or nNOS positive neurons by the number of HuC/D staining neurons for each ganglia within a HPF, and the mean proportion from each HPF was then calculated. Measurements were recorded using an Axiovert S100TV fluorescence microscope (Carl Zeiss, Thornwood, NY), and images captured using a Nikon Eclipse E400 with Digital Sight and NIS-Elements BR 3.0 digital imaging software (Nikon Canada Inc, Mississauga, ON).

### Statistical analysis

Results are expressed as means ± SD. Data were analyzed using two-way ANOVA to determine the main effects of resection (TB versus SBS) and exogenous GLP-2 (presence versus absence of GLP-2 in TPN), as well as the interaction of resection and GLP-2 together. Planned comparisons of treatment means were evaluated by unpaired t-tests. Correlations between bowel and neuronal morphology were determined using univariate linear regression. All statistical tests were performed using GraphPad Prism 6.0 statistical software (GraphPad, San Diego, CA). *P* < 0.05 was deemed statistically significant.

## Results

### Plasma hormones and glucose

**Fig 2:**
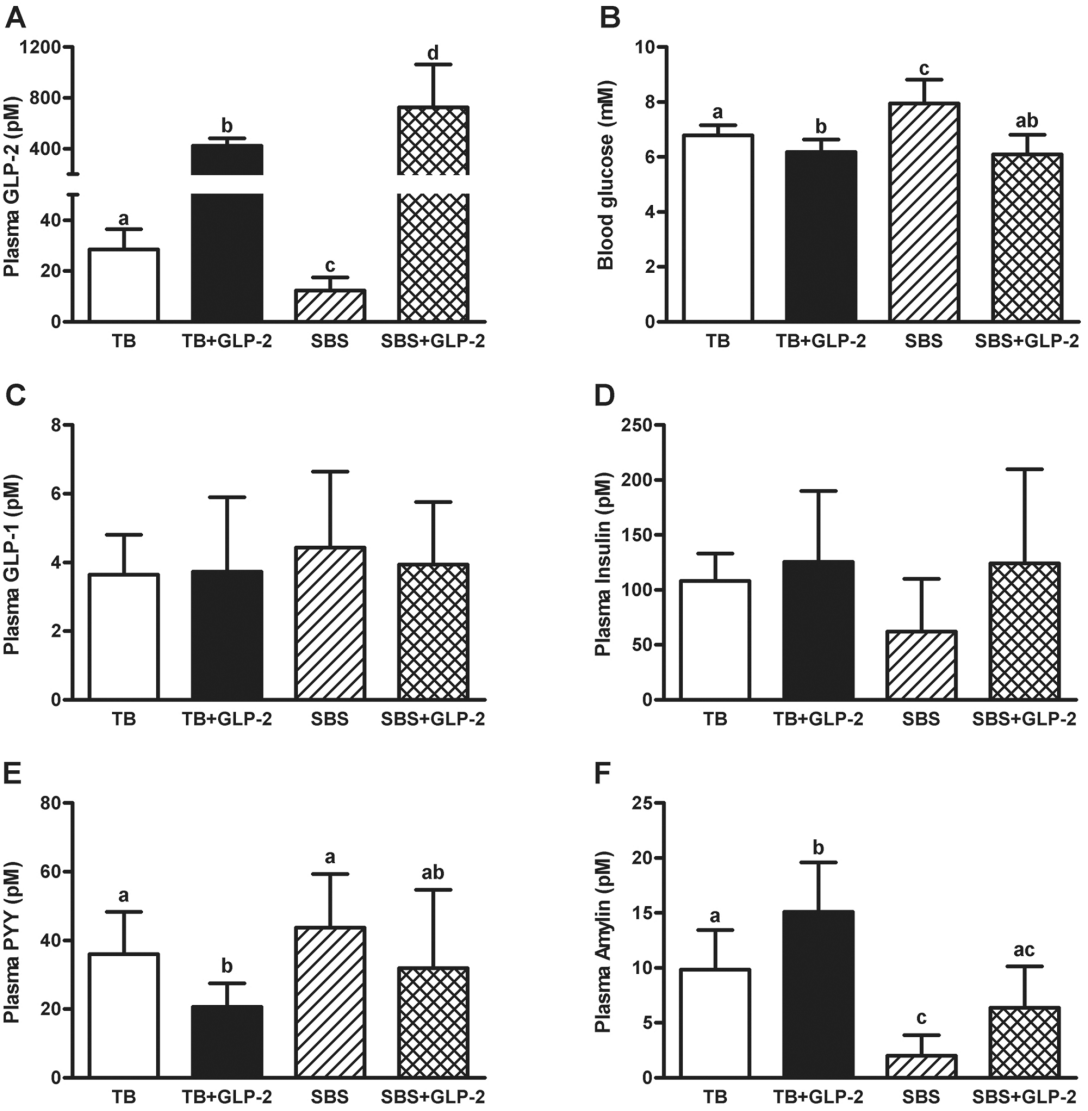
Plasma hormone and glucose concentrations. Rats were subjected to bowel transection or resection, then maintained with TPN for 7 d with or without exogenous GLP-2. The fasting plasma concentrations include GLP-2 (A), glucose (B), GLP-1 (C), insulin (D), PYY (E), and amylin (F). Values are means±SD. Labelled means with different superscripts were significantly different (*P* < 0.05). TB (n = 6–7), TB GLP-2 (n = 7), SBS (n = 3–5), SBS GLP-2 (n = 5–7).

We found a significant resection × GLP-2 interaction on plasma concentrations of GLP-2 and glucose (Fig 2, Table 2). Treatment with exogenous GLP-2 increased plasma GLP-2 concentrations in both TB and SBS groups, while bowel resection alone reduced plasma GLP-2. GLP-2 infusion decreased blood glucose concentrations in the TB groups. Bowel resection resulted in an increase in blood glucose compared to TB that was attenuated by GLP-2 treatment. The main effects of GLP-2 on plasma PYY concentrations, as well as the main effects of GLP-2 and resection on amylin, were significant by two-way ANOVA (Table 2). Exogenous GLP-2 decreased plasma PYY concentrations, but increased amylin in TB animals and had no effect on GLP-1 or insulin. Bowel resection reduced plasma amylin, but had no effect on GLP-1, insulin or PYY. The raw data for these observations and all the findings from this study can be found at https://doi.org/10.6084/m9.figshare.4832648.v1.

**Table 2:** Significance of main effects and interactions of resection with GLP-2 on all endpoints depicted in figures.

	Significance by Two-Way ANOVA (*P*-value)^1^		
	Resection	GLP-2	Resection × GLP-2
Plasma Variables			
GLP-2	0.0559	<0.0001	0.0351
Glucose	0.0427	<0.0001	0.0210
GLP-1	0.5666	0.8167	0.7408
Insulin	0.3639	0.1332	0.3876
PYY	0.1364	0.0372	0.7790
Amylin	<0.0001	0.0122	0.8017
Body Weight and Composition			
Weight gain	<0.0001	0.6278	0.2689
Fluid%	0.3827	0.7303	0.2126
Fat%	<0.0001	0.1867	0.0268
Lean%	0.0017	0.3819	0.0185
Hexose Transporter and GLP-2 Receptor mRNA			
SGLT-1	0.0841	0.1215	0.5207
GLUT-2	0.0015	0.0415	0.4076
GLUT-5	0.1305	0.5344	0.3787
GLP-2R	0.3809	0.0002	0.0003
Myenteric Plexus Immunopositivity			
Jejunum nNOS:HuC/D	0.0133	0.8381	0.7381
Colon nNOS:HuC/D	<0.0001	0.4726	0.0636

^1^Results of two-way ANOVA examining the main effect of resection (TB versus SBS), the main effect of GLP-2 (presence versus absence of GLP-2 in TPN), and interaction of resection and GLP-2 together.

### Body weight and composition

**Fig 3:**
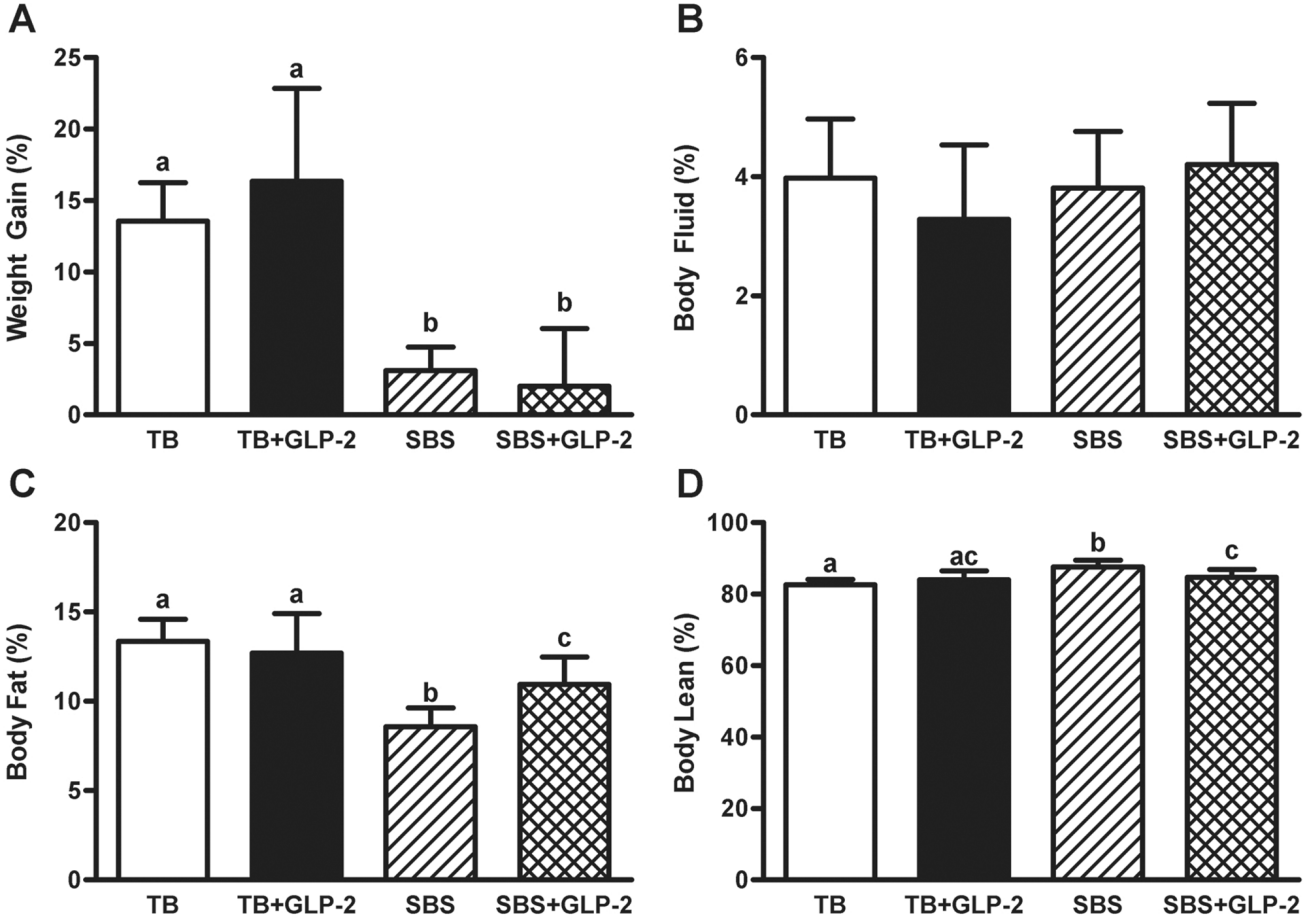
Body weight and composition. Rats were subjected to bowel transection or resection, then maintained with TPN for 7 d with or without exogenous GLP-2. Percent weight gain (A), percent body fluid (B), percent body fat (C) and percent body lean (D) composition. Values are means±SD. Labelled means with different superscripts were significantly different (*P* < 0.05). TB (n = 7), TB GLP-2 (n = 7), SBS (n = 5), SBS GLP-2 (n = 7).

There were differing effects of resection and exogenous GLP-2 on body weight and composition (Fig 3, Table 2). Bowel resection significantly decreased weight gain compared to TB groups, while exogenous GLP-2 treatment had no effect. Bowel resection resulted in a decrease in body fat percent with a reciprocal increase in lean percent composition, which were both attenuated by GLP-2 treatment. Exogenous GLP-2 alone had no impact on body composition (fluid, fat or lean percent) in TB animals.

### Bowel morphology and crypt cell proliferation

Compared to TB rats, treatment with exogenous GLP-2 increased the total pre-anastomotic jejunal weight, length and mucosal surface area, and proximal jejunal weight, villus height, crypt width, mucosal-to-serosal amplification ratio and mucosal surface area (Table 3). Resection alone increased jejunal crypt width, jejunal and colonic muscularis propria thickness, and, in GLP-2 treated animals, also increased proximal jejunal weight. Jejunal circumference and villus width, and colonic length and circumference did not change with GLP-2 treatment or resection. Bowel resection increased colon weight, and jejunal and colonic CCPI, compared to TB, while exogenous GLP-2 had no effect.

**Table 3:** Bowel morphology and crypt cell proliferation.

	Transected Bowel		Short Bowel Syndrome		Significance by Two-Way ANOVA (*P*-value)^1^		
	TB	TB GLP-2	SBS	SBS GLP-2	Resection	GLP-2	Resection × GLP-2
Total Pre-Anastomotic Jejunum							
Weight (g)	2.04±0.23^a^	3.85±0.34^b^	2.19±0.48^a^	4.03±0.24^b^	0.2084	<0.0001	0.8790
Length (cm)	25.0±2.6^a^	32.9±2.7^b^	25.7±1.8^a^	33.9±3.3^b^	0.4168	<0.0001	0.8638
Mucosal SA (cm^2^)	197.2±38.8^a^	395.8±132.9^b^	173.8±39.8^a^	405.7±99.4^b^	0.8644	<0.0001	0.6737
Proximal 10 cm Jejunum							
Weight (g)	0.83±0.09^a^	1.27±0.16^b^	0.87±0.14^a^	1.49±0.15^c^	0.0244	<0.0001	0.0983
Circumference (mm)	9±1	10±1	9±2	10±1	0.8054	0.0119	0.5510
Villus Height (μm)	355±89^a^	610±178^b^	361±82^a^	597±102^b^	0.9490	0.0001	0.8557
Villus Width (μm)	106±15	120±9	103±27	119±26	0.8505	0.0880	0.8974
Crypt Width (μm)	32±2^a^	39±5^b^	40±5^b^	40±5^b^	0.0341	0.0632	0.0991
Mucosal:Serosal Amplification Ratio	8.3±1.8^a^	12.0±3.4^b^	7.9±2.1^a^	11.9±3.0^b^	0.8346	0.0031	0.9209
Mucosal SA (cm^2^)	77.4±15.5^a^	120.4±36.5^b^	67.8±16.1^a^	119.3±24.0^b^	0.6244	0.0003	0.6962
Muscularis Propria Thickness (μm)	84±22^a^	105±32^ab^	132±19^b^	136±22^b^^#^	0.0010	0.2293	0.4173
CCPI	0.14±0.07^a^	0.21±0.06^ab^	0.25±0.06^bc^	0.28±0.06^c^	0.0011	0.0631	0.5136
Colon							
Weight (g)	1.08±0.10^a^	1.09±0.14^a^	1.42±0.15^b^	1.46±0.16^b^	<0.0001	0.6059	0.7761
Length (cm)	11.9±1.1	12.4±1.5	12.5±1.4	12.2±0.9	0.7159	0.8270	0.4261
Circumference (mm)	14±1	14±2	15±3	12±2	0.7008	0.0645	0.1337
Muscularis Propria Thickness (μm)	116±33^a^	119±20^a^	162±35^b^	149±25^b^	0.0027	0.6351	0.4729
CCPI	0.04±0.04^a^	0.03±0.01^a^	0.11±0.08^b^	0.14±0.04^b^	<0.0001	0.8173	0.3522

Morphometry and CCPI data for jejunum (total small bowel distal to ligament of Treitz and proximal to anastomosis [= total pre-anastomotic jejunum] and 10 cm length of small bowel distal to ligament of Treitz [= proximal 10 cm jejunum]) and colon (from ascending colon to rectum) derived from the following sample sizes: TB (n = 5–7), TB GLP-2 (n = 6–7), SBS (n = 4–5), SBS GLP-2 (n = 7). CCPI was calculated from the number of BrdU positive nuclei / total nuclei per crypt. Values are means±SD. Labelled means with different superscripts were significantly different (*P* < 0.05).

^#^ denotes *P* = 0.06 versus TB GLP-2.

^1^Results of two-way ANOVA examining the main effect of resection (TB versus SBS), the main effect of GLP-2 (presence versus absence of GLP-2 in TPN), and interaction of resection and GLP-2 together.

Abbreviations: surface area (**SA**).

### qPCR

**Fig 4:**
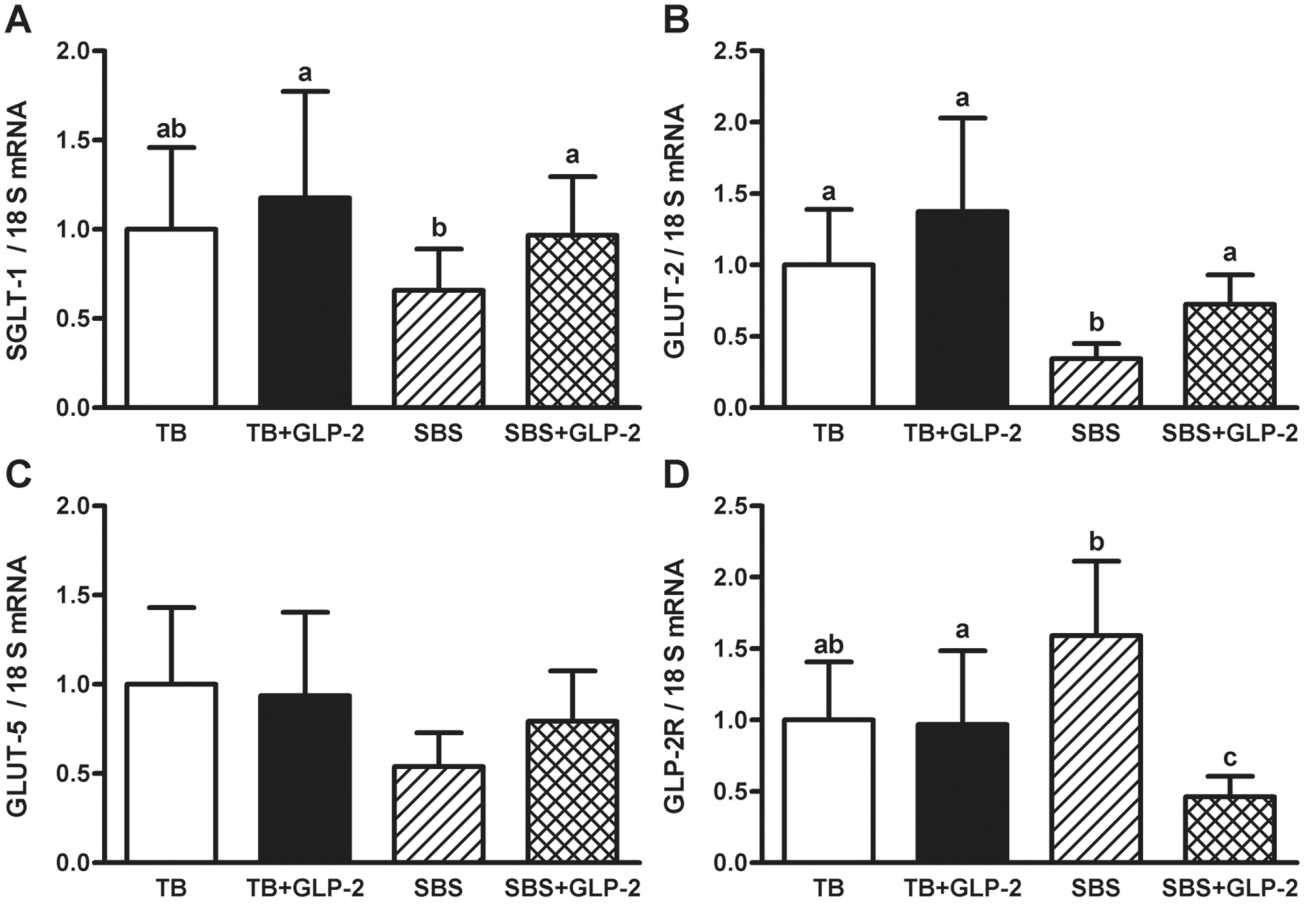
Jejunal hexose transporter and GLP-2R transcript abundance. Rats were subjected to bowel transection or resection, then maintained with TPN for 7 d with or without exogenous GLP-2. SGLT-1 (A), GLUT-2 (B), GLUT-5 (C) and GLP-2R (D) mRNA abundance by qPCR. Data were expressed as relative fold changes using 18S ribosomal RNA as the endogenous control. Values are means, with error bars representing the upper and lower limits. Labelled means with different superscripts were significantly different (*P* < 0.05). TB (n = 7), TB GLP-2 (n = 7), SBS (n = 5), SBS GLP-2 (n = 7).

There was a significant main effect of resection and GLP-2 on GLUT-2 mRNA and a significant interaction of resection and GLP-2 on GLP-2R mRNA in the jejunum (Table 2). Exogenous GLP-2 increased SGLT-1 and GLUT-2 mRNA abundance only in resected animals. Resection lowered mRNA abundance of GLUT-2 compared to TB (Fig 4). Although exogenous GLP-2 alone had no effect on jejunal GLP-2R transcript abundance in TB, it decreased GLP-2R mRNA in SBS groups. There were no statistically significant differences in GLUT-5 between the groups.

### ENS morphology

**Fig 5:**
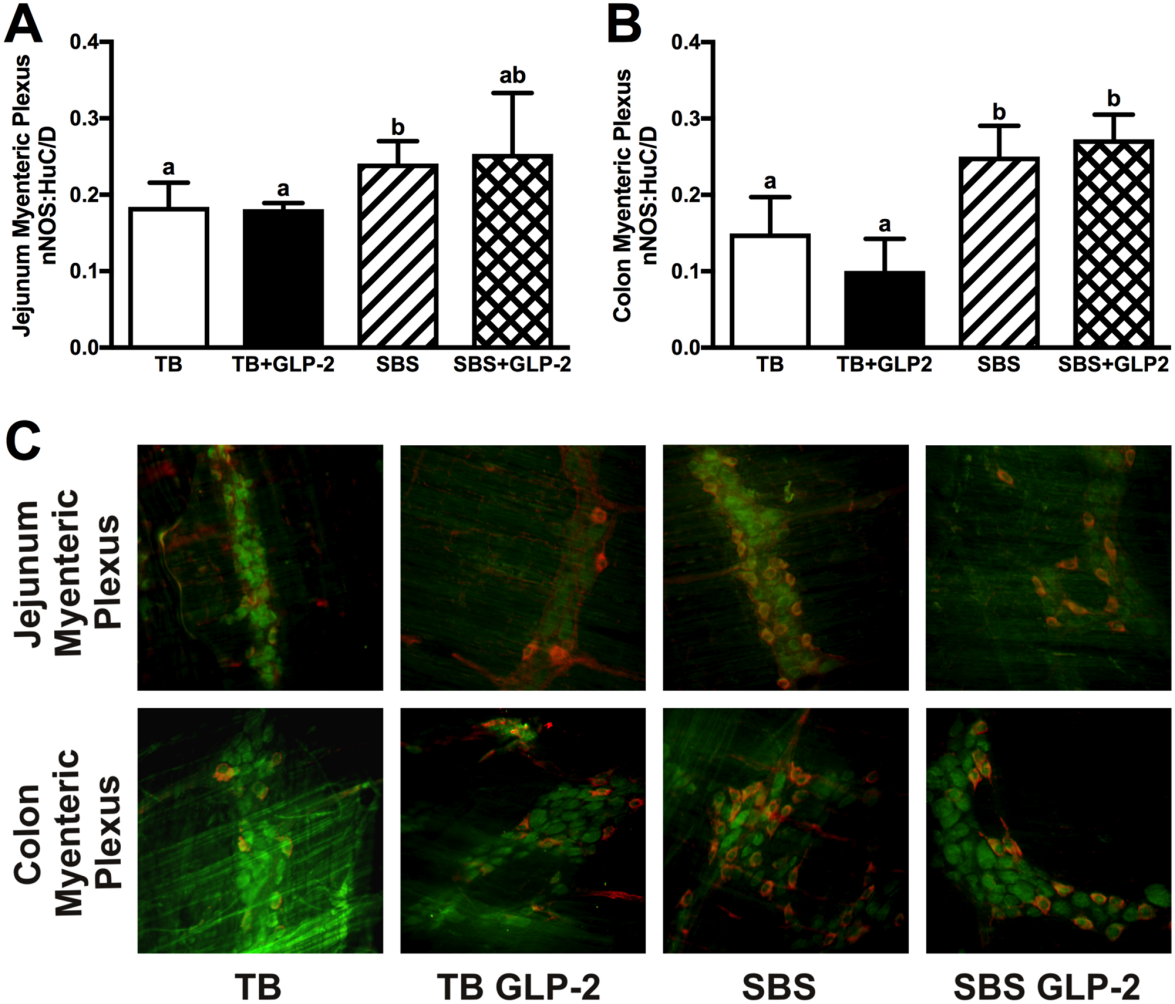
Whole mount immunohistochemistry. Rats were subjected to bowel transection or resection, then maintained with TPN for 7 d with or without exogenous GLP-2. Proportion of nNOS staining neurons (number of nNOS positive neurons / total HuC/D neuronal nuclear stained neurons per ganglia) in jejunal (A) and colonic (B) myenteric plexuses. Representative whole-mount immunohistochemistry for anti-HuC/D (neuronal nuclei stain in green) and anti-nNOS (perinuclear stain in red) at 20X magnification (C). Values are means±SD. Labelled means with different superscripts were significantly different (*P* < 0.05). TB (n = 4), TB GLP-2 (n = 4), SBS (n = 5), SBS GLP-2 (n = 5).

**Fig 6:**
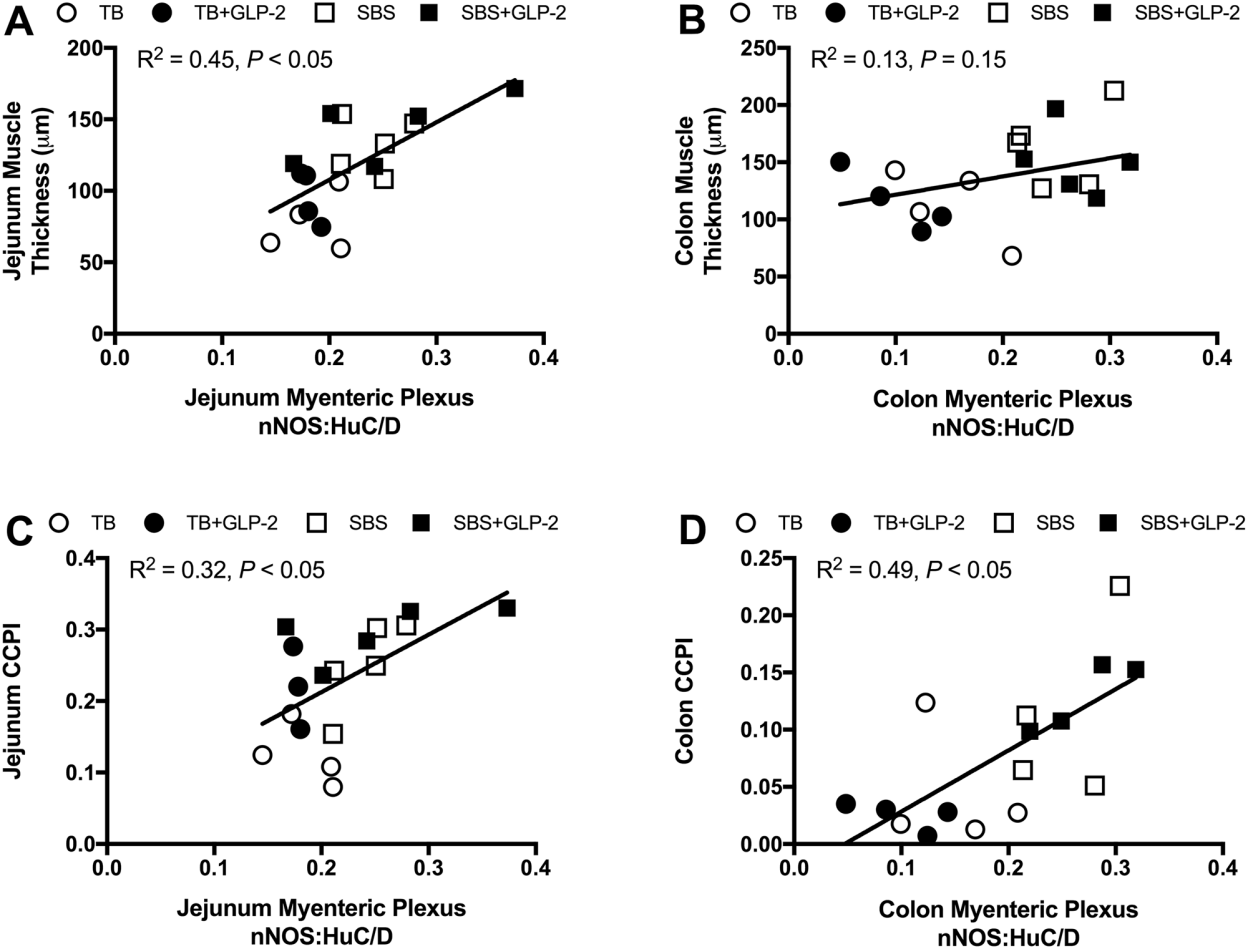
Muscularis propria thickness and crypt cell proliferation correlate with myenteric nNOS immunopositivity. Rats were subjected to bowel transection or resection, then maintained with TPN for 7 d with or without exogenous GLP-2. Scatterplots of muscularis propria thickness versus proportion of myenteric nNOS immunopositive neurons (number of nNOS positive neurons / total HuC/D neurons per ganglia) in the jejunum (A) and colon (B). Scatterplots of CCPI (number of BrdU positive nuclei / total nuclei per crypt) versus proportion of myenteric nNOS immunopositive neurons in the jejunum (C) and colon (D). TB (white circle), TB GLP-2 (black circle), SBS (white square), SBS GLP-2 (black square). Univariate linear regression line plotted with associated R^2^ value, with *P* < 0.05 representing statistical significance.

Bowel resection increased the proportion of neurons that were costained for nNOS and HuC/D in jejunal and colonic myenteric plexuses (Fig 5, Table 2), but not in the submucosal plexus. Exogenous GLP-2 treatment had no effect on nNOS and HuC/D immunopositivity. There were no differences in size and density of ganglia, nor proportions of immunopositive VIP neurons, with resection or exogenous GLP-2. Muscularis propria thickness correlated with myenteric nNOS:HuC/D immunopositivity in the jejunal myenteric plexus, with a non-statistically significant correlation in the colon (Fig 6A and 6B). Jejunal and colonic CCPI were positively associated with myenteric nNOS:HuC/D immunopositivity (Fig 6C and 6D).

## Discussion

In this study, we have shown that there were different effects of massive distal resection and exogenous GLP-2 on intestinal and systemic adaptive responses. Massive distal resection, but not exogenous GLP-2, increased jejunal and colonic muscularis propria thickness, crypt cell proliferation and myenteric nNOS immunopositivity. Moreover, we have provided evidence that resection-induced reductions in transcript abundance of hexose transporters and increases in GLP-2R in remnant jejunum were attenuated by exogenous GLP-2 therapy. We also demonstrated that resection and exogenous GLP-2 exert divergent effects on plasma concentrations of amylin and PYY. And, finally, we have reported for the first time that exogenous GLP-2 attenuates resection-induced alterations in adipose reserves and blood glucose concentrations. Together, these results suggest that exogenous GLP-2 exerts metabolic effects beyond its well-characterized intestinotrophic outcomes.

Exogenous GLP-2 had more potent intestinotrophic effects compared to bowel resection alone, increasing small bowel weight, length, villus height, crypt width and mucosal surface area. As jejunal CCPI remained unchanged, the trophic effects of exogenous GLP-2 infusion may have been due to reduced apoptosis [32]. Isolated bowel resection had fewer effects on small bowel morphology, consistent with reports describing inadequate native jejunal adaptation after massive ileocecal resection [33, 34]. This was in contrast to what we and others have demonstrated previously, noting striking increases in jejunal villus height or width, mucosal weight, DNA and protein content, and maltase activity after massive distal resection [23, 35]. Discrepancies in findings were likely due to differences in extent of resection, location of transection in TB groups, the site of jejunal sampling and enteral nutrition. In the current study, 60% of the intestine was resected, encompassing a substantial portion of the jejunum, the entire ileum and cecum, while jejunal specimens were sampled from the proximal 10 cm near the duodenojejunal flexure, remote from the anastomosis (40 cm away). Our study minimised the effect of bowel transection on endpoints compared to other reports describing tissue collection closer to the anastomotic site (1 to 3 cm away) [23, 35]. Other studies have shown that the magnitude of post-resection adaptation was greater adjacent to the anastomosis, potentially due to inflammation and neovascularization, leading to an influx of oxygen and nutrients [36, 37]. Moreover, it is possible that this may have also been related to stronger paracrine stimulation by endogenous GLP-2 from remnant colon, whereas more proximal bowel may have relied to a greater extent on endocrine effects of GLP-2. In support of this, we observed that proximal segments of jejunum express higher abundance of GLP-2R mRNA compared to distal segments closer to the anastomosis [38]. An additional contrasting feature between studies includes the enteral intake of the animals—our previous report involved rats who were fed, instead of being fasted as in the current experiment [23]. Differences in the response to distal resection between studies may have reflected the effect of fasting on mucosal atrophy and luminal nutrition on intestinal adaptation [2].

Along with an increase in morphological indices of mucosal growth, there also appeared to be stimulation of functional adaptation following exogenous GLP-2 treatment. Jejunal SGLT-1 transcript level was upregulated by exogenous GLP-2 in SBS, and resection-induced decreases in GLUT-2 mRNA abundance were attenuated by GLP-2 infusion. The lack of effects of exogenous GLP-2 on hexose transporters in TB rats might reflect a ceiling of mRNA transcription in this model. Unexpectedly, these transporters were upregulated in GLP-2 treated SBS animals despite a decrease in GLP-2R mRNA abundance. It remains to be determined whether changes in transcript levels are reflected in protein content of hexose transporters and GLP-2R, and whether the intestinotrophic effects of exogenous GLP-2 are mediated through extra-intestinal mechanisms.

This study explored the relationships between post-resection adaptation, exogenous GLP-2 and the ENS. It is interesting to note that in the resected groups, muscularis propria thickness and crypt cell proliferation correlated with myenteric nNOS immunopositivity. nNOS is normally distributed in the myenteric plexus and plays a major role in inhibiting intestinal motility [39, 40]. Increased proportions of myenteric nNOS seen after resection in our experiments is suggestive of a compensatory mechanism to reduce motility and improve absorption of nutrients and water. Further, the correlation of nNOS with crypt cell kinetics and muscle thickness was consistent with other reports demonstrating a regulatory role for nitric oxide and the myenteric plexus in jejunal crypt cell proliferation [41, 42]. Although we did not detect any changes in the ratio of VIP staining neurons, previous studies have described inhibitory and stimulatory effects of proximal and mid bowel resection on VIP abundance in the ENS [43, 44]. Inconsistencies in results could be due to the absence of transected bowel controls, or the effect of location and extent of resection on specific neuroeffectors [43–46]. Interestingly, neuronal responses evoked by intestinal resection were not recapitulated by exogenous GLP-2 infusion. Systemic exogenous GLP-2 had no effect on any neuronal parameter measured, including the density of ganglia, neurons per ganglia, and abundance of VIP and nNOS, which was in contrast to the stimulatory effects of exogenous GLP-2 on VIP and nNOS immunopositivity under *in vitro* conditions [20]. Although our observations were limited to morphological ENS endpoints, it appears that alterations in VIP and nNOS neuronal expression were unlikely to be mediators of the intestinotrophic effects of exogenous GLP-2. Our findings were consistent with previous studies in VIP knock-out mice detailing the VIP-independent trophic effects of GLP-2 [47].

We found reduced percent body fat and increased lean percent in TPN fed rats 7 d after bowel resection, consistent with other reports using enterally fed SBS mice after 14 d [48, 49]. Although statistically significant, it is unclear whether this increase in percent lean composition with resection is clinically relevant or whether this is a dilutional effect of losing fat at a greater rate while preserving lean mass. It is also unknown whether these changes in body composition persist in the long-term. Importantly, we found that GLP-2 attenuated the reduction in percent fat mass produced by SBS at study termination. These findings resemble other reports in SBS patients wherein GLP-2 together with GLP-1, but not alone, increased adipose mass in the short-term [21]. Our study was amongst the most detailed reports of the effects of exogenous GLP-2 and bowel resection on secretion of other gut hormones. Previous models have identified multiple growth factors, including insulin-like growth factor-1, keratinocyte growth factor, epidermal growth factor and related molecules, as key mediators of intestinal adaptation [50–54]. However, the role of other gut hormones released by enteroendocrine L-cells, including GLP-1 and PYY, have not been well studied [55, 56]. Proglucagon mRNA and plasma GLP-2 concentrations vary depending on the SBS resection model in rats [34, 57, 58]. In our model with massive resection of a majority of the jejunum and the entire ileocecum, we observed an isolated reduction in plasma GLP-2 without alterations in GLP-1 or PYY levels after 7 d; it remains to be determined whether differential elimination rates of these L-cell products contribute to their plasma concentrations. Moreover, exogenous GLP-2 therapy decreased PYY levels without affecting GLP-1 concentrations, suggestive of divergent negative feedback on L-cells.

Resection and exogenous GLP-2 produced reciprocal effects on concentration of plasma glucose and pancreatic amylin. Exogenous GLP-2 decreased resection-induced hyperglycemia in an insulin-independent manner. In piglets maintained with TPN, GLP-2 infusion has been shown to increase glucose uptake and metabolism by portal drained viscera [8]. Moreover, there is evidence of GLP-2R expression in murine mesenteric fat [59]. Alternatively, previous studies have demonstrated that intravenous GLP-2 increases peripheral glucose clearance and insulin sensitivity in wild-type mice, but not in brain specific pro-opiomelanocortin-GLP-2R knock-outs [14]. Taken together, these studies indicate that tissue metabolic responses with exogenous GLP-2 could be due to a direct GLP-2R-mediated effect on viscera and adipose tissue, or via central GLP-2R-dependent signaling mechanisms. Consistent with the known role of amylin analogues as hypoglycemic agents in diabetes mellitus, reduced plasma amylin in the current study may have played a role in resection-induced hyperglycemia and worsening of peripheral glucose clearance [60]. Our data demonstrated that exogenous GLP-2 is an amylin secretagogue, and suggested that both hormones may facilitate peripheral glucose disposal and decrease plasma glucose concentrations together. Although there were no changes in plasma insulin levels despite known co-secretion with amylin, differences in insulin concentration followed a similar pattern to plasma amylin between the groups, yet did not reach statistical significance [61, 62]. However, lack of significant effects of exogenous GLP-2 infusion on plasma insulin is consistent with previous reports in humans with SBS [21]. Previous studies investigating the impact of exogenous GLP-1 on plasma amylin and insulin in diabetic rats have shown that differential responses in the magnitude of amylin and insulin secretion are also possible [61]. Although infusion of GLP-2 in humans with SBS does not affect overnight fasting or post-prandial blood glucose levels, our results indicated that exogenous GLP-2 and bowel resection may modulate glucose homeostasis beyond their well-described intestinotrophic outcomes in rats maintained on TPN [21].

In summary, we demonstrated the following: (1) massive distal resection led to an increased proportion of nNOS immunopositive myenteric neurons, thicker intestinal muscularis propria and crypt cell proliferation which were not recapitulated by exogenous GLP-2 therapy; (2) intestinotrophic effects of exogenous GLP-2 were apparent, but were unlikely to be associated with alterations in VIP and nNOS expression in the ENS; (3) exogenous GLP-2 attenuated resection-related changes in body composition; (4) exogenous GLP-2 and resection led to reciprocal effects on amylin (increased by GLP-2, decreased by resection); and (5) exogenous GLP-2 may have improved glycemic control and attenuated resection-induced increases in blood glucose concentrations. Thus, our findings indicated that exogenous GLP-2 and massive distal bowel resection exert disparate intestinal and extra-intestinal manifestations that modulate body composition over the short-term with clinical implications for patients with SBS. Further studies with a longer duration of treatment may provide more insight into the impact (and underlying mechanisms) of bowel resection and GLP-2 on the ENS and systemic metabolism, including their effects on glucose and lipid metabolism in peripheral tissues.
